# Association of cumulative methylprednisolone dosages with mortality risk from pneumonia in connective tissue disease patients

**DOI:** 10.1038/s41598-024-78233-5

**Published:** 2024-11-03

**Authors:** Saibin Wang, Qian Ye

**Affiliations:** 1 Department of Pulmonary and Critical Care Medicine, Jinhua Municipal Central Hospital, No. 365, East Renmin Road, Jinhua, 321000 Zhejiang Province China; 2 School of Medicine, Shaoxing University, Shaoxing, 312000 Zhejiang Province China; 3 Department of Medical Records Quality Management, Jinhua Municipal Central Hospital, Jinhua, 321000 Zhejiang Province China

**Keywords:** Glucocorticoids, Methylprednisolone, Cumulative doses, Community-acquired pneumonia, Mortality, Immunology, Diseases, Rheumatology

## Abstract

Corticosteroid is essential in the treatment regimen for connective tissue disease (CTD); however, its long-term use poses significant risks, including pulmonary infections. The relationship between cumulative corticosteroid doses and adverse pneumonia outcomes requires further investigation. This study aimed to explore the association between cumulative methylprednisolone dosages (CMD) and pneumonia mortality risks among CTD patients. We conducted a retrospective analysis of data from CTD patients treated with intravenous or oral corticosteroids across six academic hospitals over approximately five years in China. We evaluated follow-up outcomes at 30 and 90 days after the onset of pneumonia. Piecewise linear regression, Cox regression analysis, and survival analysis were employed to investigate the relationship between CMD and 30-day and 90-day mortality risks. Among 335 CTD patients with pneumonia, the mean CMD was 12 g. The 30-day and 90-day mortality rates were 25.07% and 29.55%, respectively. After adjusting for potential confounders, smooth curve fitting analysis revealed a specific nonlinear relationship between CMD and 30-day and 90-day mortality risks. Cox regression analysis indicated that the lowest pneumonia mortality risk occurred when CMD ranged from 11 to 24 g (30-day adjusted hazard ratio (aHR) 0.33, 95% CI 0.14–0.77; 90-day aHR 0.37, 95% CI 0.17–0.81). Patients in the 11–24 g CMD group demonstrated significantly lower cumulative hazard and death rates compared to both the low CMD (< 11 g) and high CMD (> 24 g) groups (*P* < 0.05). Furthermore, interaction testing suggested that CMD’s negative impact on pneumonia mortality risks was more pronounced in community-acquired pneumonia (CAP) compared to hospital-acquired pneumonia (*P* for interaction < 0.05). CMD shows a distinct nonlinear relationship with 30-day and 90-day pneumonia mortality risks, with potentially lower risks observed within the 11–24 g CMD range. Moreover, the varying impact of CMD on CAP mortality risk warrants further consideration in clinical management strategies.

**Supplementary Information:**

The online version contains supplementary material available at 10.1038/s41598-024-78233-5.

## Introduction

Connective tissue disease (CTD) encompass a group of autoimmune disorders characterized by chronic inflammation of the connective tissue, affecting multiple organs and systems within the body^1^. The therapeutic arsenal for CTD includes nonsteroidal anti-inflammatory drugs (NSAIDs), glucocorticoids, immunosuppressants, and biologics^2^. Among these, glucocorticoids such as prednisone and methylprednisolone play a pivotal role in the management of CTD^2,3^. They exert potent anti-inflammatory and immunosuppressive effects, positioning them as frontline therapies for various rheumatic diseases^3,4^.

Despite their efficacy, prolonged or high-dose glucocorticoid use is associated with significant adverse effects, including infections, hypertension, and osteoporosis^5^. Of particular concern is the immunosuppressive nature of glucocorticoids, which compromises the body’s defense against pathogens^6^. During high-dose treatment regimens, patients often experience sustained lymphocyte depletion, predisposing them to infections, notably pulmonary infections^4,7^. The risk of infection due to immunosuppression is therefore a critical challenge in the therapeutic landscape of CTD.

The dosage and duration of glucocorticoid therapy are closely linked to the risk of infections^3,5^. Accumulative glucocorticoid exposure merits further investigation regarding its correlation with the risk of pulmonary infections and mortality in CTD patients. In this study, we investigated the correlation between cumulative methylprednisolone dosages (CMD) and pneumonia mortality risk in patients with CTD. Our objective is to offer insights into optimizing glucocorticoid dosing to reduce the mortality risk associated with pneumonia while maximizing therapeutic efficacy in CTD patients.

## Methods

### Study design and subjects

The study utilized case data obtained from the public repository www.Datadryad.org^8^. The study population consisted of 716 hospitalized patients treated with intravenous or oral steroids for underlying conditions such as CTD, nephrotic syndrome, chronic glomerulonephritis, or idiopathic interstitial pneumonia, diagnosed with pneumonia upon admission or during hospitalization between 2013 and 2017 at six academic hospitals in China^9^. Pneumonia diagnosis followed criteria outlined by the American Thoracic Society and the Infectious Disease Society of America^10,11^.

**Fig. 1:**
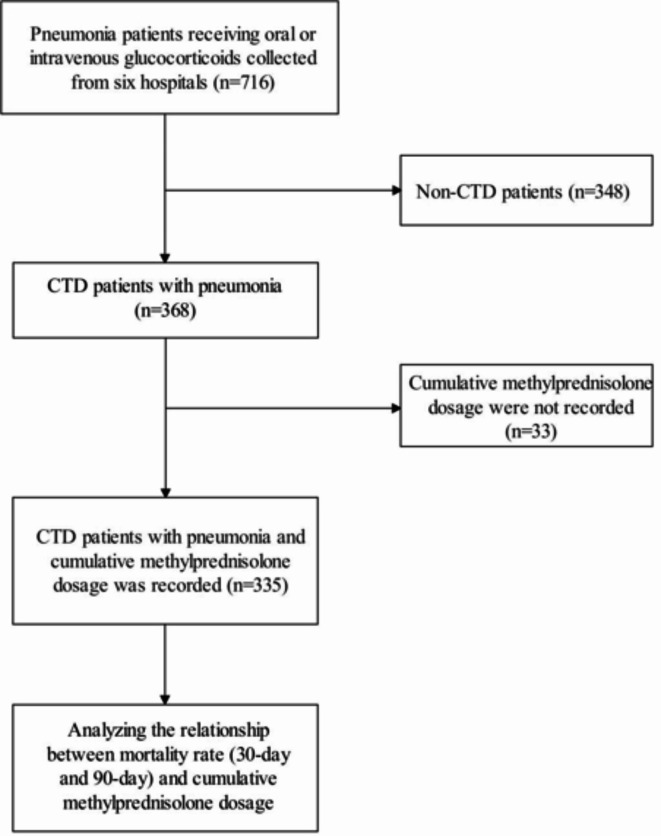
Study flow chart.

From this cohort, a targeted population of 335 patients diagnosed with CTD was identified through a screening process outlined in Fig. 1. Inclusion criteria were: (a) adults aged 18 years and older diagnosed with CTD and pneumonia receiving intravenous or oral steroid therapy, (b) availability of microbiological testing data from sputum or bronchoalveolar lavage, and (c) complete cumulative dose records of methylprednisolone use. Exclusion criteria included cases without pneumonia diagnosis and those lacking informed consent.

We confirm that all methods were carried out in accordance with relevant guidelines and regulations. The reporting of this study conforms to the Strengthening the Reporting of Observational Studies in Epidemiology (STROBE) statement^12^. Ethical approval for this retrospective study was granted by the Ethics Committee of the China–Japan Friendship Hospital, facilitating centralized collaboration and approval across all participating institutions^9^.

## Data collection

Detailed demographic and clinical data were systematically gathered for analysis. Demographic variables encompassed gender distribution and age stratification (> 60 years). Smoking history was also documented. Pre-existing conditions were meticulously recorded, including incidences of interstitial lung disease (ILD), hypertension, coronary heart disease, diabetes mellitus, anemia, chronic renal failure, cerebrovascular disease, tumors, chronic obstructive pulmonary disease, bronchiectasis, and persistent lymphocytopenia. Pneumonia characteristics such as CURB-65 score ≥ 2 and community-acquired status were documented alongside etiological factors, encompassing Cytomegalovirus, Pneumocystis, Aspergillus, Influenza A virus, Influenza B virus, Respiratory syncytial virus, Herpes simplex virus type 1, Adenovirus, Humanrhinovirus, Mycobacterium tuberculosis, Haemophilus influenzae, Enterobacter cloacae, Burkholderia, Enterococcus, Stenotrophomonas, Escherichia coli, Comamonas acidovorans, Legionella, Pseudomonas, Acinetobacter, and Klebsiella pneumoniae. Treatment interventions detailed antibiotic regimens, ganciclovir usage, anti-Aspergillus therapies, sulfanilamide administration, extracorporeal membrane oxygenation (ECMO), continuous veno-venous hemofiltration (CVVH), oxygen supplementation, mechanical ventilation, vasoactive medications, and immunosuppressive protocols. Laboratory parameters included white blood cell count, neutrophil and lymphocyte counts, hemoglobin, platelet counts, aspartate aminotransferase, alanine aminotransferase, blood urea nitrogen, serum creatinine, potassium, sodium, and procalcitonin levels^9^.

### Statistical analysis

The statistical methods employed in this study included descriptive analyses for both categorical and continuous data. Categorical data were presented as frequencies (percentages), while continuous data were expressed as medians (interquartile ranges, IQRs). Missing values were handled using multiple imputation techniques. Intergroup comparisons of categorical variables, Pearson’s chi-squared tests or Fisher’s exact tests were applied as appropriate. The relationship between CMD and 30-day and 90-day mortality risks was examined using a generalized additive model with smoothing curve fitting and piecewise linear regression analysis. Kaplan-Meier analysis with the log-rank test was employed to assess the association between different CMD levels and the risks of 30-day and 90-day mortality. Cox regression analysis was conducted to evaluate the specific effects of CMD on 30-day and 90-day mortality risks, adjusting for covariates identified through a covariate-discrimination algorithm. Two distinct criteria were employed for covariate selection: (1) Criteria I: A covariate-discrimination algorithm was used, where variables were included if their addition or removal resulted in a ≥ 10% change in the regression coefficient. Covariates meeting this criterion included ILD, anemia, cerebrovascular diseases, oxygen inhalation, and procalcitonin. (2) Criteria II: Clinically relevant variables such as age, gender, and the use of other immunosuppressants were adjusted based on clinical considerations.

All statistical analyses were performed using R software (version 3.5.1). A P-value < 0.05 was considered statistically significant.

## Results

### Mortality and pneumonia characteristics

Of the 335 CTD with pneumonia, the 30-day mortality rate was 25.07% (84/335), and the 90-day mortality rate was 29.55% (99/335). Within the cohort, 38.21% were male, and 51.94% were aged over 60 years. The most prevalent comorbidities included ILD in 61.49%, hypertension in 35.52%, and diabetes mellitus in 25.07%.

Regarding pneumonia types and treatment, community-acquired pneumonia (CAP) was predominant (91.64%, 307 patients), with a CURB-65 score ≥ 2 observed in 28.96% of cases. The etiology of pneumonia varied, with Cytomegalovirus identified in 14.93%, Pneumocystis in 7.76%, and Aspergillus in 5.67%. Mixed etiologies, involving two or more of the aforementioned pathogens, accounted for 11.64%. Pre-hospitalization antibiotic therapy was administered in 64.18% of cases. Immunosuppressants were used in 72.84% of cases. The median CMD was 5.4 g (IQR 2.40–13.59 g). In critical care interventions, ventilation was required in 38.51% of cases, and vasoactive drugs in 17.07%. ECMO and CVVH were utilized in 4.48% and 10.15% of cases, respectively. Further demographic characteristics and laboratory findings are detailed in Table 1.

**Table 1:** Clinical characteristics of CTD patients with pneumonia.

Characteristics	Values
30-day mortality, n (%)	84 (25.07)
90-day mortality, n (%)	99 (29.55)
Male, n (%)	128 (38.21)
Age > 60 years, n (%)	174 (51.94)
Smoke, n (%)	56 (16.72)
*Combined diseases*,* n (%)*	
ILD	206 (61.49)
Hypertension	119 (35.52)
CHD	42 (12.54)
Diabetes mellitus	84 (25.07)
Anemia	28 (8.36)
CRF	16 (4.78)
Cerebrovascular disease	21 (6.27)
Tumor	11 (3.28)
COPD	19 (5.67)
Bronchiectasia	13 (3.88)
Persistent lymphocytopenia	149 (44.48)
*Pneumonia and treatment*,* n (%)*	
CURB-65 score ≥ 2	97 (28.96)
Community-acquired pneumonia	307 (91.64)
Etiology	
Cytomegalovirus	50 (14.93)
*Pneumocystis*	26 (7.76)
*Aspergillus*	19 (5.67)
Mixed etiologies*	39 (11.64)
Other pathogens**	85 (25.37)
Non-identified	116 (34.63)
Multidrug-resistant bacteria	47(14.03)
Antibiotics	215 (64.18)
Ganciclovir use	147 (43.88)
Anti-*Aspergillus*	130 (38.81)
Sulfanilamide use	147 (43.88)
ECMO	15 (4.48)
CVVH	34 (10.15)
Oxygen inhalation	255 (76.12)
Ventilation	129 (38.51)
Vasoactive drugs	57 (17.07)
Immunosuppressant^#^	244 (72.84)
Cumulative methylprednisolone dosages (g), median (IQR)	5.40(2.40-13.59)
Duration of methylprednisolone use (month), median (IQR)	6.00 (2.00-31.50)
*Blood test*,* median (IQR)*	
White blood cell (×10^9^/L)	7.78(5.72–11.05)
Neutrophils (×10^9^/L)	6.34(4.31–9.43)
Lymphocyte (×10^9^/L)	0.81(0.46–1.39)
Hemoglobin, g/L	112(97.00-128.00)
Platelet counts (×10^9^/L)	180(124.00-251.00)
AST, U/L	25 (17.00–43.00)
ALT, U/L	24 (15.00–49.00)
BUN, mmol/L	5.75(4.37–8.38)
Serum creatinine, mmol/L	60.1(47.45-83.00)
Potassium (mmol/L)	3.9 (3.60–4.20)
Sodium (mmol/L)	138(135.00-140.85)
Procalcitonin (ng/mL)	0.44(0.15–4.76)

*Contains two or more of the three pathogens Pneumocystis, Cytomegalovirus, and Aspergillus.

**Other pathogens refer to single or mixed infections, including the following pathogens: Influenza A virus, Influenza B virus, Respiratory syncytial virus, Herpes simplex virus type 1, Adenovirus, Humanrhinovirus, Mycobacterium tuberculosis, Haemophilus influenzae, Enterobacter cloacae, Burkholderia, Enterococcus, Stenotrophomonas, Escherichia coli, Comamonas acidovorans, and Legionella, Pseudomonas, Acinetobacter, and Klebsiella pneumoniae.

^#^Immunosuppressants include methotrexate, cyclosporine, cyclophosphamide, tacrolimus, sirolimus, and azathioprine.

CTD, connective tissue disease; ILD, interstitial lung disease; CHD, coronary heart disease; CRF, chronic renal failure; COPD, chronic obstructive pulmonary disease; ECMO, extracorporeal membrane oxygenation; CVVH, continuous venovenous hemofiltration; AST, aspartate aminotransferase; ALT, alanine aminotransferase; BUN, blood urea nitrogen.

### Relationship between CMD and mortality risk

**Fig. 2:**
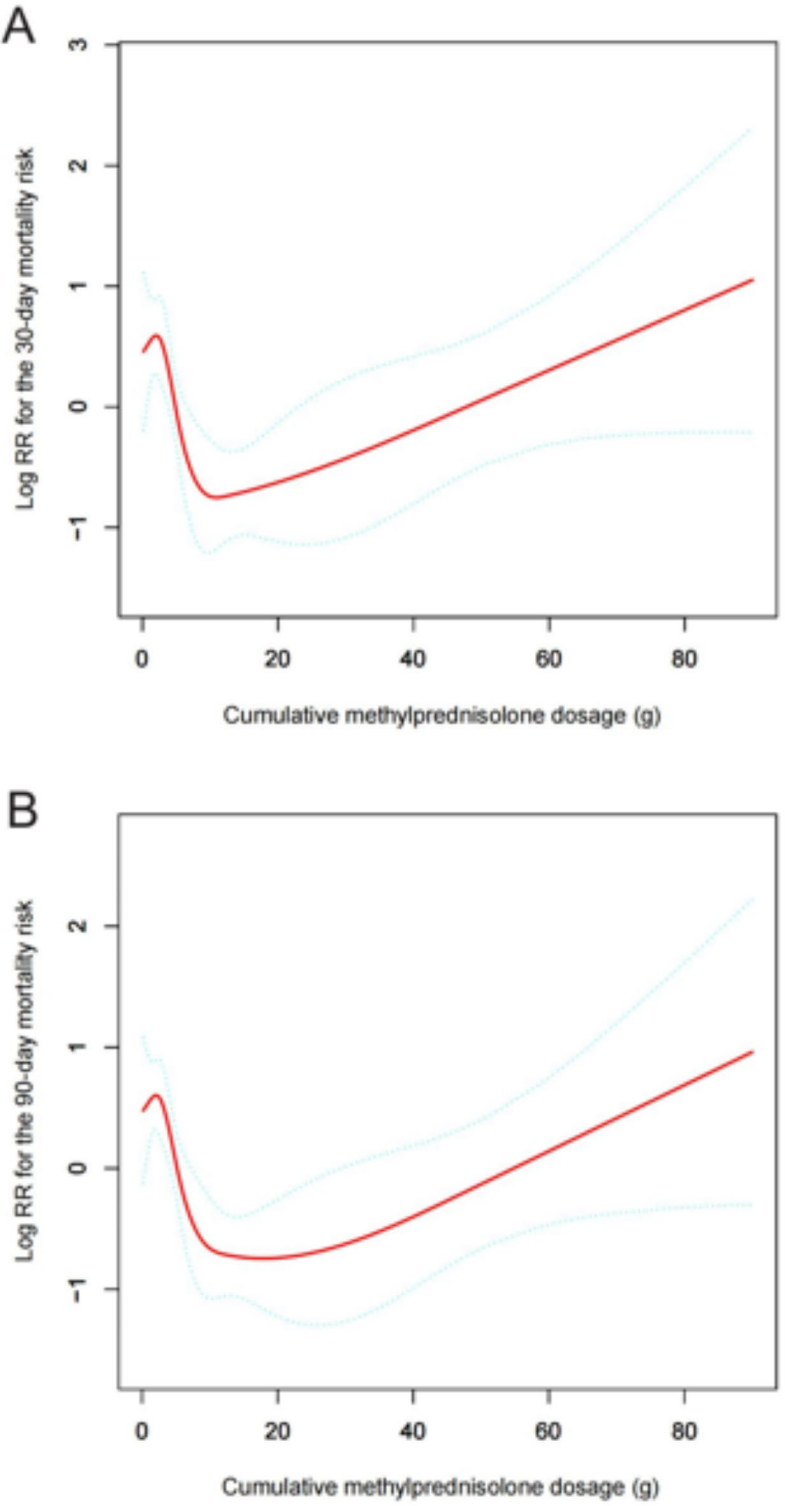
Smooth fitting curve illustrating the association between cumulative methylprednisolone dosages and (**A**) 30-day and (**B**) 90-day mortality risk in CTD patients with pneumonia. Adjustments were made for age, gender, immunosuppressants, interstitial lung disease, anemia, cerebrovascular diseases, oxygen inhalation, and procalcitonin. CTD, connective tissue disease.

After adjusting for confounding variables (age, gender, immunosuppressants, ILD, anemia, cerebrovascular diseases, oxygen inhalation, and procalcitonin), the smoothed curve fitting revealed a distinctive “tick-shaped” relationship between CMD and 30-day and 90-day mortality risks (Fig. 2). As CMD increased, mortality risk decreased gradually to a nadir and then significantly increased thereafter.

## Identification of optimal dosage

**Fig. 3:**
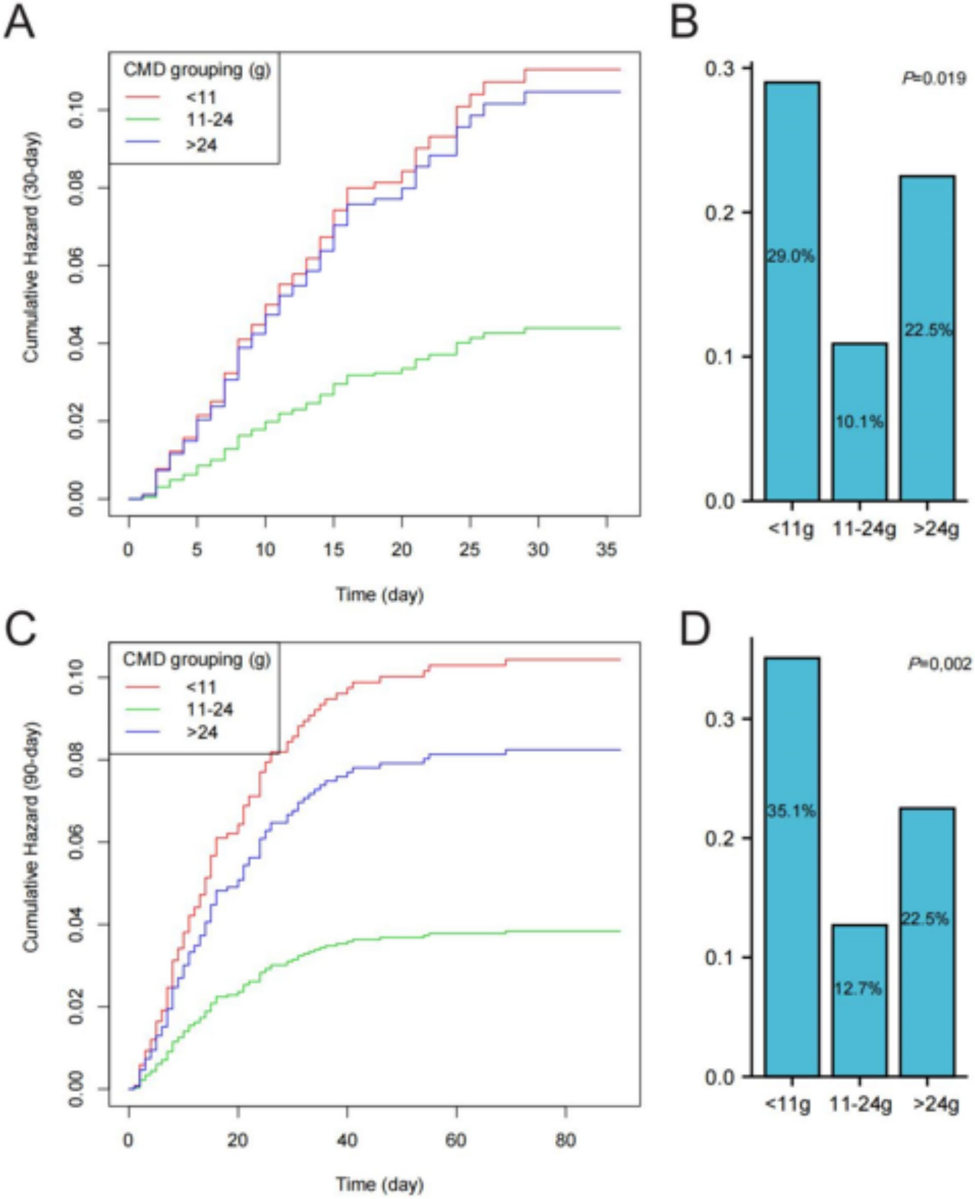
Kaplan-Meier analysis and comparison of mortality rates across different levels of cumulative methylprednisolone dosages (< 11 g, 11–24 g, and > 24 g). Panels show (**A**) 30-day cumulative hazard curve, (**B**) comparison of 30-day mortality rates, (**C**) 90-day cumulative hazard curve, and (**D**) comparison of 90-day mortality rates. During Kaplan-Meier analysis, adjustments were made for age, gender, immunosuppressants, interstitial lung disease, anemia, cerebrovascular diseases, oxygen inhalation, and procalcitonin. CMD, cumulative methylprednisolone dosages.

Given the observed “tick-shaped” relationship in the smoothed curve fitting between CMD and 30-day, 90-day mortality risks, we further investigated the dosage range where mortality risk was minimized, pinpointing it at a CMD of 18 g (confidence interval [CI] 11–24 g) (Table 2). Subsequently, stratifying CMD into three groups with 11 g and 24 g as the threshold values, we conducted Cox regression analysis. Compared to other dosage groups (< 11 g, and > 24 g), the 11–24 g group exhibited the lowest 30-day (adjusted hazard ratio [aHR] 0.33, 95% CI 0.14–0.77) and 90-day (aHR 0.37, 95% CI 0.17–0.81) mortality risks (Table 3). Moreover, Fig. 3 illustrates the comparison of 30-day and 90-day mortality risks among patients receiving different CMD, demonstrating that the group within the 11–24 g range experienced the lowest cumulative hazard and mortality rates (*P* < 0.05).

**Table 2:** Threshold effect of cumulative methylprednisolone dosage on the risk of mortality in CTD patients with pneumonia.

Outcome	30-day mortality	90-day mortality
*Model I*	Adjusted HR (95% CI) P-value	
A straight line effect	1.00 (0.98, 1.01) 0.5749	0.99 (0.97, 1.01) 0.2178
*Model II*		
Inflection point (g)	18	18
<18 effect	0.91 (0.86, 0.96) 0.0003	0.90 (0.86, 0.95) < 0.0001
>18 effect	1.03 (1.01, 1.06) 0.0020	1.03 (1.01, 1.05) 0.0025
HR between < 18 and > 18	1.14 (1.06, 1.22) 0.0002	1.15 (1.07, 1.22) < 0.0001
Log-likelihood ratio	< 0.001	< 0.001

Adjust for: age, gender, immunosuppressants (methotrexate, cyclosporine, cyclophosphamide, tacrolimus, sirolimus and azathioprine), procalcitonin, interstitial lung disease, anemia, cerebrovascular diseases, and oxygen inhalation.

**Table 3:** Cox regression analysis of cumulative methylprednisolone dosage on the risk of mortality in CTD patients with pneumonia.

Exposure	Model I: non-adjusted	Model II: adjusted*
Cumulative methylprednisolone dosage grouping (g)	30-day mortality, HR (95% CI) P-value	
< 11	1.0	1.0
11–24	0.34 (0.15, 0.78) 0.0111	0.33 (0.14, 0.77) 0.0102
>24	0.74 (0.39, 1.40) 0.3544	0.75 (0.39, 1.45) 0.3913
Cumulative methylprednisolone dosage grouping (g)	90-day mortality, HR (95% CI) P-value	
< 11	1.0	1.0
11–24	0.32 (0.15, 0.69) 0.0038	0.37 (0.17, 0.81) 0.0134
>24	0.60 (0.32, 1.13) 0.1168	0.79 (0.41, 1.51) 0.4749

*Adjust for: age, gender, immunosuppressants (methotrexate, cyclosporine, cyclophosphamide, tacrolimus, sirolimus and azathioprine), procalcitonin, interstitial lung disease, anemia, cerebrovascular diseases, and oxygen inhalation.

## Interaction analysis on CAP

**Fig. 4:**
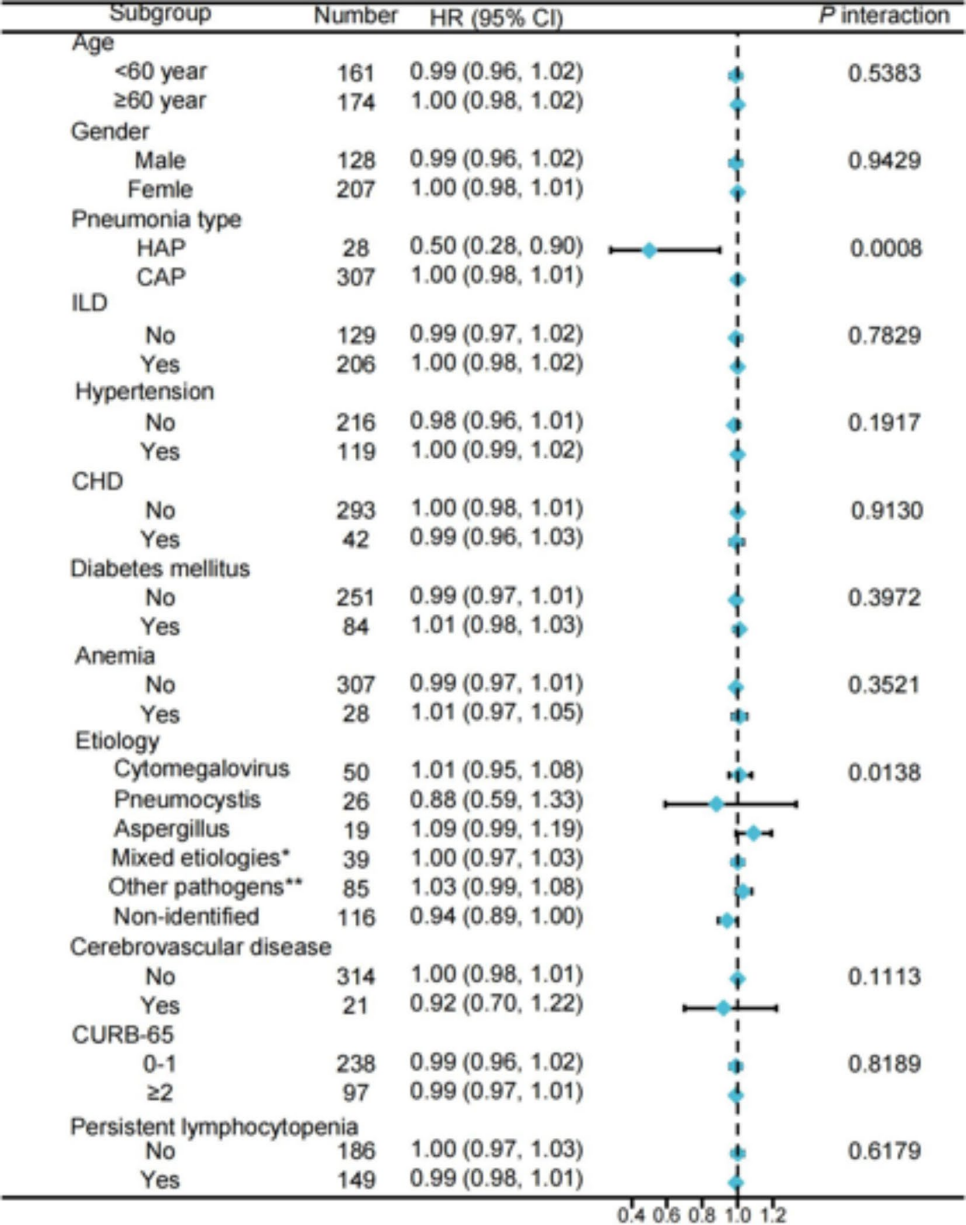
Subgroup analysis of the impact of cumulative methylprednisolone dosages on 30-day mortality risk in CTD patients with pneumonia. Adjustments were made for age, gender, immunosuppressants, ILD, anemia, cerebrovascular diseases, oxygen inhalation, and procalcitonin, except where the variable was analyzed. CTD, connective tissue disease; HAP, hospital-acquired pneumonia; CAP, community-acquired pneumonia; ILD, interstitial lung disease; CHD, coronary heart disease.

An intriguing finding emerged from interaction testing, indicating that the detrimental impact of increased CMD on 30-day and 90-day mortality risks was more pronounced in patients with CAP compared to those without (Fig. 4, Supplementary Fig. 1, *P* for interaction < 0.05).

## Discussion

Our study identifies a distinctive “tick-shaped” relationship between CMD and 30-day and 90-day mortality risks among CTD patients with pneumonia. Specifically, we observed significantly higher mortality risks associated with pneumonia when CMD levels fell below 11 g or exceeded 24 g, compared to levels between 11 g and 24 g. Additionally, the detrimental effect of CMD on pneumonia mortality risk was more pronounced in CAP patients compared to those with hospital-acquired pneumonia (HAP).

Glucocorticoids are fundamental in managing CTD by attenuating inflammation in diseases such as systemic lupus erythematosus and rheumatoid arthritis, but their use is associated with heightened susceptibility to pulmonary infections^2,13^. Pathogens commonly implicated in pneumonia secondary to prolonged glucocorticoids use include Pneumocystis jirovecii, Cytomegalovirus, and Aspergillus, which complicate treatment and contribute to increased mortality rates^14–17^.

Balancing the therapeutic benefits of glucocorticoids against their infection-related risks is pivotal in clinical decision-making^18,19^. Previous studies consistently report that long-term glucocorticoid therapy correlates with elevated infection risks across multiple organ systems, often dose-dependent in severity^13,19,20^. The recommendation for glucocorticoids therapy in pneumonia varies depending on the type and severity of the pneumonia: while mild cases generally do not require glucocorticoids, severe forms such as acute respiratory distress syndrome or pneumonia leading to respiratory failure may benefit from their administration^21–23^. In the present study, we identified a distinctive “tick-shaped” relationship between CMD and 30-day and 90-day mortality risks, where mortality risk initially decreased with increasing CMD but subsequently rose significantly. While direct literature on this specific pattern is lacking, indirect evidence provides some insights. Previous study indicates that nearly all cells are sensitive to glucocorticoid regulation^24^, and glucocorticoid effects can be prolonged^18^. Glucocorticoid plays a complex role in infectious diseases; appropriate glucocorticoid therapy can reduce systemic inflammation, improve lung and extrapulmonary organ function, shorten mechanical ventilation duration, and decrease ICU stay, potentially lowering mortality rates^25–27^. However, excessive glucocorticoid use can increase mortality risks, particularly from pneumonia^28,29^. This intricate balance between the beneficial and detrimental effects of glucocorticoid therapy may account for the observed “tick-shaped” pattern in mortality risk. Furthermore, our findings introduce a novel perspective by revealing a non-linear relationship between CMD and infection-related mortality risks. The identified threshold effect suggests that moderate CMD levels (11–24 g) may mitigate the mortality risks associated with infections in CTD patients.

Our study specifically focused on pneumonia within the CTD population, many of whom were undergoing long-term steroid treatment. We differentiated between CAP and HAP, revealing varying mortality risks between these subgroups. In discussing the varying mortality risks between CAP and HAP within the CTD population, especially among those on long-term steroid treatment, several mechanisms may be considered. The variation in pathogens between CAP and HAP may contribute to differences in disease severity and treatment complexity. HAP pathogens were more likely to exhibit multi-drug resistance, which complicated treatment and may exacerbate outcomes. Additionally, HAP patients, who were often hospitalized and had multiple underlying health conditions, were at a higher risk of severe infections and may respond less favorably to treatment compared to those with CAP^30^. Particularly noteworthy was the amplified negative impact of CMD on mortality risk observed in CAP patients. This finding may help reconcile previous conflicting reports in the literature, where some studies suggested increased mortality with glucocorticoid use, while others indicated improved prognosis or found no significant benefit^29,31,32^. Such discrepancies likely stem from differences in pneumonia type, disease severity, and individual patient characteristics.

### Limitations

Some limitations of our study are noteworthy. Firstly, our study design primarily relied on retrospective data, inevitably introducing biases. Secondly, our investigation exclusively focused on methylprednisolone without exploring other steroids such as prednisone or dexamethasone, thus limiting the generalizability of our findings regarding the relationship between different glucocorticoids and mortality risk. Lastly, pathogens were not detected in some patients until at least 48 h after admission, potentially increasing the risk of hospital-acquired infections. Additionally, detailed data on specific Gram-positive and Gram-negative bacteria could not be presented due to the limitations of the public database used, which lacks detailed bacterial classifications. Despite these limitations, our study’s findings contribute novel insights into the understanding of the relationship between steroid use and mortality in CTD patients with pneumonia.

## Conclusions

Our research reveals a unique association between CMD levels and mortality risk among CTD patients with pneumonia, indicating a potential impact of CMD levels on pneumonia prognosis, especially in cases of CAP. Given the possible ramifications of this association, careful consideration is advised when tailoring glucocorticoid treatment strategies for CTD patients. Future research should further elucidate optimal dosing strategies to maximize therapeutic benefits while minimizing infection-related risks in this vulnerable patient population.

## Electronic supplementary material

Below is the link to the electronic supplementary material.

Supplementary Material 1

Supplementary Material 2

Supplementary Material 3

Supplementary Material 4

## Data Availability

The original contributions presented in the study are included in the article/supplementary material, further inquiries can be directed to the corresponding author.

## Abbreviations

CTD: Connective tissue disease
CMD: Cumulative methylprednisolone dosages
CAP: Community-acquired pneumonia
ILD: Interstitial lung disease
ECMO: Extracorporeal membrane oxygenation
CVVH: Continuous veno-venous hemofiltration

## References

[1] Mathai, S. C. & Danoff, S. K. Management of interstitial lung disease associated with connective tissue disease. *BMJ*. **352**, h6819 (2016). 26912511 10.1136/bmj.h6819 PMC6887350

[2] Smolen, J. S. et al. EULAR recommendations for the management of rheumatoid arthritis with synthetic and biological disease-modifying antirheumatic drugs: 2019 update. *Ann. Rheum. Dis.* **79** (6), 685–699 (2020). 31969328 10.1136/annrheumdis-2019-216655

[3] Buttgereit, F. et al. Standardised nomenclature for glucocorticoid dosages and glucocorticoid treatment regimens: current questions and tentative answers in rheumatology. *Ann. Rheum. Dis.* **61** (8), 718–722 (2002). 12117678 10.1136/ard.61.8.718 PMC1754188

[4] Rhen, T. & Cidlowski, J. A. Antiinflammatory action of glucocorticoids–new mechanisms for old drugs. *N Engl. J. Med.* **353** (16), 1711–1723 (2005). 16236742 10.1056/NEJMra050541

[5] Liu, D. et al. A practical guide to the monitoring and management of the complications of systemic corticosteroid therapy. *Allergy Asthma Clin. Immunol.* **9** (1), 30 (2013). 23947590 10.1186/1710-1492-9-30 PMC3765115

[6] Pitre, T. et al. Corticoglucocorticoids in Community-Acquired Bacterial Pneumonia: a systematic review, pairwise and dose-response Meta-analysis. *J. Gen. Intern. Med.* **38** (11), 2593–2606 (2023). 37076606 10.1007/s11606-023-08203-6 PMC10115386

[7] RECOVERY Collaborative Group. Higher dose corticoglucocorticoids in patients admitted to hospital with COVID-19 who are hypoxic but not requiring ventilatory support (RECOVERY): a randomised, controlled, open-label, platform trial. *Lance*. **401** (10387), 1499–1507 (2023). 10.1016/S0140-6736(23)00510-X PMC10156147 37060915

[8] Li, L. et al. Aetiology and prognostic risk factors of mortality in pneumonia patients receiving glucocorticoids alone or glucocorticoids and other immunosuppressants: a retrospective cohort study [Dataset]. Dryad. (2020). 10.5061/dryad.mkkwh70x2. Accessed 15 Jun 2024. 10.1136/bmjopen-2020-037419 PMC7592294 33109645

[9] Li, L. et al. Aetiology and prognostic risk factors of mortality in patients with pneumonia receiving glucocorticoids alone or glucocorticoids and other immunosuppressants: a retrospective cohort study. *BMJ Open.* **10** (10), e037419 (2020). 33109645 10.1136/bmjopen-2020-037419 PMC7592294

[10] Sousa, D. et al. Community-acquired pneumonia in immunocompromised older patients: incidence, causative organisms and outcome. *Clin. Microbiol. Infect.* **19** (2), 187–192 (2013). 22390624 10.1111/j.1469-0691.2012.03765.x

[11] American Thoracic Society; Infectious Diseases Society of America. Guidelines for the management of adults with hospital-acquired, ventilator-associated, and healthcare-associated pneumonia. *Am. J. Respir Crit. Care Med.* **171** (4), 388–416 (2005). 15699079 10.1164/rccm.200405-644ST

[12] Vandenbroucke, J. P. et al. Strengthening the reporting of Observational studies in Epidemiology (STROBE): explanation and elaboration. *PLoS Med.* **4** (10), e297 (2007). 17941715 10.1371/journal.pmed.0040297 PMC2020496

[13] Strehl, C. et al. Defining conditions where long-term glucocorticoid treatment has an acceptably low level of harm to facilitate implementation of existing recommendations: viewpoints from an EULAR task force. *Ann. Rheum. Dis.* **75** (6), 952–957 (2016). 26933146 10.1136/annrheumdis-2015-208916

[14] Fanouriakis, A. et al. EULAR recommendations for the management of systemic lupus erythematosus: 2023 update. *Ann. Rheum. Dis.* **83** (1), 15–29 (2024). 37827694 10.1136/ard-2023-224762

[15] Di Matteo, A., Bathon, J. M. & Emery, P. Rheumatoid arthritis. *Lancet*. **402** (10416), 2019–2033 (2023). 38240831 10.1016/S0140-6736(23)01525-8

[16] Zhao, Z., Huang, Y., Ming, B., Zhong, J. & Dong, L. Characterization and associated risk factors of Pneumocystis Jirovecii pneumonia in patients with AIRD: a retrospective study. *Rheumatol. (Oxford)*. **61** (9), 3766–3776 (2022). 10.1093/rheumatology/keab941 34962999

[17] Agustí, C. et al. Pulmonary infiltrates in patients receiving long-term glucocorticoid treatment: etiology, prognostic factors, and associated inflammatory response. *Chest*. **123** (2), 488–498 (2003). 12576371 10.1378/chest.123.2.488

[18] Seguro, L. P., Rosario, C. & Shoenfeld, Y. Long-term complications of past glucocorticoid use. *Autoimmun. Rev.* **12** (5), 629–632 (2013). 23261815 10.1016/j.autrev.2012.12.002

[19] Chastain, D. B., Spradlin, M., Ahmad, H. & Henao-Martínez, A. F. Unintended consequences: risk of opportunistic infections Associated with Long-term glucocorticoid therapies in adults. *Clin. Infect. Dis.* **78** (4), e37–e56 (2024). 37669916 10.1093/cid/ciad474

[20] Barbulescu, A., Sjölander, A., Delcoigne, B., Askling, J. & Frisell, T. Glucocorticoid exposure and the risk of serious infections in rheumatoid arthritis: a marginal structural model application. *Rheumatol. (Oxford)*. **62** (10), 3391–3399 (2023). 10.1093/rheumatology/kead083 PMC10547528 36821426

[21] Peng, B. et al. Clinical value of glucocorticoids for severe community-acquired pneumonia: a systematic review and meta-analysis based on randomized controlled trials. *Med. (Baltim).* **102** (46), e36047 (2023). 10.1097/MD.0000000000036047 PMC10659673 37986401

[22] Wu, D., Li, Y., Dong, S. H. & Gao, Y. Clinical outcomes of corticosteroid administration for acute respiratory distress syndrome in adults based on meta-analyses and trial sequential analysis. *Ann. Saudi Med.* **44** (3), 167–182 (2024). 38853475 10.5144/0256-4947.2024.167 PMC11268472

[23] Bru, J. P. The role of systemic corticosteroids when treating infections in adult primary care. *Infect. Dis. Now*. **54** (4S), 104925 (2024). 38768709 10.1016/j.idnow.2024.104925

[24] Quatrini, L. & Ugolini, S. New insights into the cell- and tissue-specificity of glucocorticoid actions. *Cell. Mol. Immunol.* **18** (2), 269–278 (2021). 32868909 10.1038/s41423-020-00526-2 PMC7456664

[25] Pinzón, M. A. et al. Dexamethasone vs methylprednisolone high dose for Covid-19 pneumonia. *PLoS One*. **16** (5), e0252057 (2021). 34033648 10.1371/journal.pone.0252057 PMC8148307

[26] Annane, D. et al. Guidelines for the diagnosis and management of critical illness-related corticosteroid insufficiency (CIRCI) in critically ill patients (part I): society of critical Care Medicine (SCCM) and European Society of Intensive Care Medicine (ESICM) 2017. *Intensive Care Med.* **43** (12), 1751–1763 (2017). 28940011 10.1007/s00134-017-4919-5

[27] Meduri, G. U., Annane, D., Chrousos, G. P., Marik, P. E. & Sinclair, S. E. Activation and regulation of systemic inflammation in ARDS: rationale for prolonged glucocorticoid therapy. *Chest*. **136** (6), 1631–1643. 10.1378/chest.08-2408 (2009). 19801579 10.1378/chest.08-2408

[28] RECOVERY Collaborative Group. Higher dose corticosteroids in patients admitted to hospital with COVID-19 who are hypoxic but not requiring ventilatory support (RECOVERY): a randomised, controlled, open-label, platform trial. *Lancet*. **401** (10387), 1499–1507 (2023). 37060915 10.1016/S0140-6736(23)00510-X PMC10156147

[29] Tang, Q., Chen, Q., Li, Y. & Wang, Z. Association between glucocorticoids and Mortality in patients with severe pneumonia: a systematic review and Meta-analysis based on randomized controlled trials. *Comput. Math. Methods Med.* **2022**, 1191205 (2022). 35979047 10.1155/2022/1191205 PMC9377960

[30] Lanks, C. W., Musani, A. I. & Hsia, D. W. Community-acquired Pneumonia and Hospital-acquired Pneumonia. *Med. Clin. North. Am.* **103** (3), 487–501 (2019). 30955516 10.1016/j.mcna.2018.12.008

[31] Sun, L. L. et al. Meta-analysis of the clinical efficacy and safety of high- and low-dose methylprednisolone in the treatment of children with severe Mycoplasma Pneumoniae Pneumonia. *Pediatr. Infect. Dis. J.* **39** (3), 177–183 (2020). 31738328 10.1097/INF.0000000000002529

[32] Soo, C. I. et al. High-dose pulse methylprednisolone vs. dexamethasone standard therapy for severe and critical COVID-19 pneumonia: efficacy assessment in a retrospective single-centre experience from Malaysia. *Med. J. Malaysia*. **79** (1), 15–20 (2024). 38287752

