# Supplementary figures and images for: Association of cumulative methylprednisolone dosages with mortality risk from pneumonia in connective tissue disease patients
Source: Sci Rep. 2024 Nov 3;14:26502. doi: 10.1038/s41598-024-78233-5 (PMC11532547; doi:10.1038/s41598-024-78233-5)

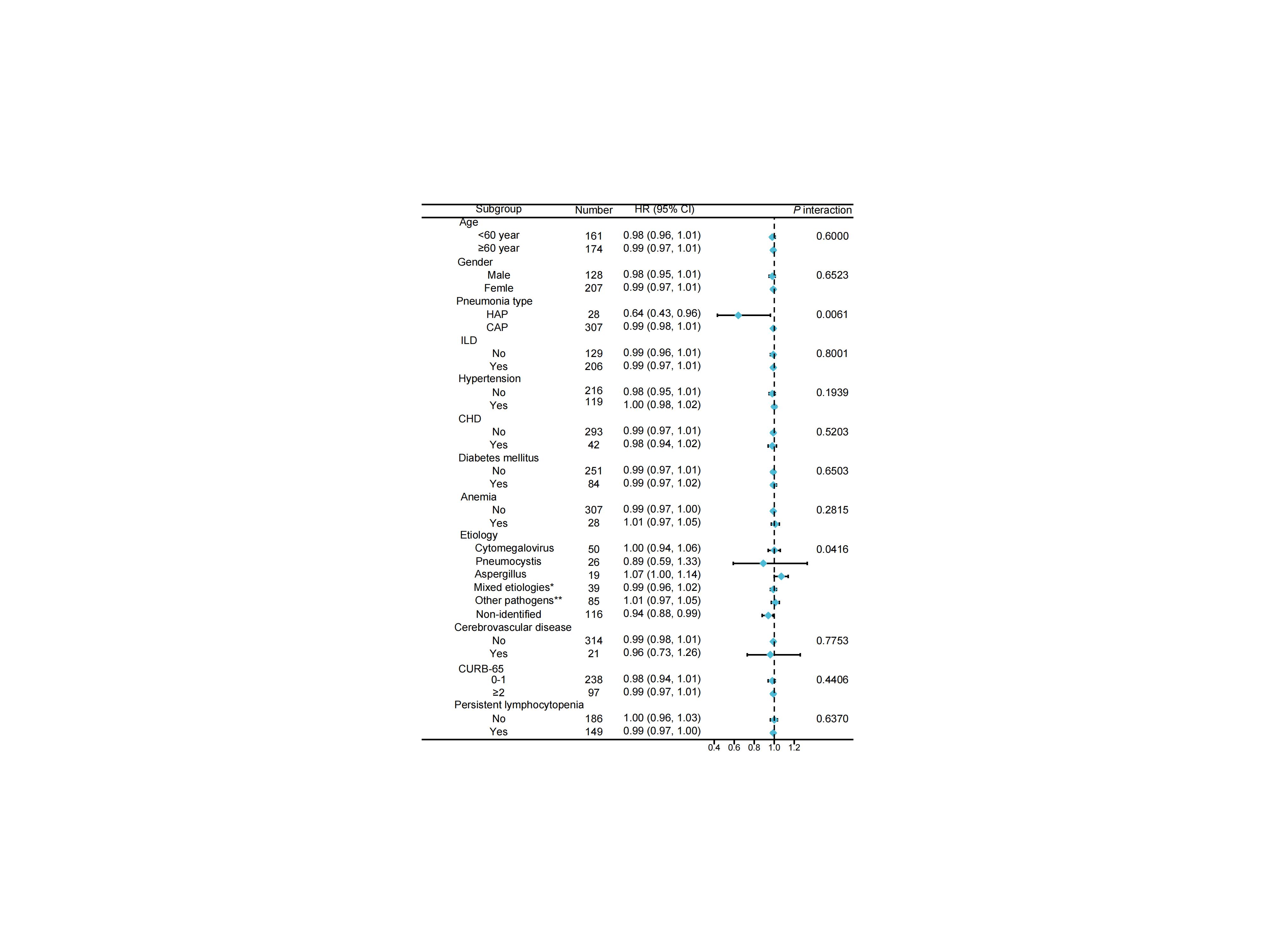

Supplement: Supplementary file 2 — Supplementary Material 2 [file 41598_2024_78233_MOESM2_ESM.jpg]
