# Supplementary material for: Association of cumulative methylprednisolone dosages with mortality risk from pneumonia in connective tissue disease patients
Source: Sci Rep. 2024 Nov 3;14:26502. doi: 10.1038/s41598-024-78233-5 (PMC11532547; doi:10.1038/s41598-024-78233-5)
Supplement: Supplementary file 3 — Supplementary Material 3 [file 41598_2024_78233_MOESM3_ESM.docx]

Figure legend

Supl. Figure 1 Subgroup analysis of the impact of cumulative methylprednisolone dosages on 90-day mortality risk in CTD patients with pneumonia. Adjustments were made for age, gender, immunosuppressants, ILD, anemia, cerebrovascular diseases, oxygen inhalation, and procalcitonin, except where the variable was analyzed. CTD, connective tissue disease; HAP, hospital-acquired pneumonia; CAP, community-acquired pneumonia; ILD, interstitial lung disease; CHD, coronary heart disease.
